# Influence of Environment and Lifestyle on Incidence and Progress of Amyotrophic Lateral Sclerosis in A German ALS Population

**DOI:** 10.14336/AD.2018.0327

**Published:** 2019-04-01

**Authors:** Sonja Korner, Johanna Kammeyer, Antonia Zapf, Magdalena Kuzma-Kozakiewicz, Maria Piotrkiewicz, Bożenna Kuraszkiewicz, Hanna Goszczynska, Marta Gromicho, Julian Grosskreutz, Peter M. Andersen, Mamede de Carvalho, Susanne Petri

**Affiliations:** ^1^Department of Neurology, Hannover Medical School, Germany.; ^2^Department of Medical Statistics, University Medical Center Göttingen Germany.; ^3^Department of Neurology, Medical University of Warsaw, Poland.; ^4^Nalecz Institute of Biocybernetics and Biomedical Engineering, Polish Academy of Sciences, Warsaw, Poland.; ^5^Institute of Physiology-Instituto de Medicina Molecular, Faculty of Medicine, University of Lisbon, Portugal; ^6^Department of Neurology, University Hospital Jena, Jena, Germany.; ^7^Department of Pharmacology and Clinical Neuroscience, Umea University, Sweden.; ^8^Center for Systems Neuroscience (ZSN), Hannover, Germany.

**Keywords:** ALS, environment, life-style, epidemiology, phenotypes

## Abstract

Amyotrophic lateral sclerosis (ALS) is a neurodegenerative disease mainly affecting upper and lower motor neurons in the brain and spinal cord. Pathogenesis of ALS is still unclear, and a multifactorial etiology is presumed. The remarkable clinical heterogeneity between different phenotypes of ALS patients suggests that environmental and lifestyle factors could play a role in onset and progression of ALS. We analyzed a cohort of 117 ALS patients and 93 controls. ALS patients and controls were compared regarding physical activity, dietary habits, smoking, residential environment, potentially toxic environmental factors and profession before symptom onset and throughout the disease course. Data were collected by a personal interview. For statistical analysis descriptive statistics, statistical tests and analysis of variance were used. ALS patients and controls did not differ regarding smoking, diet and extent of physical training. No higher frequency of toxic influences could be detected in the ALS group. ALS patients lived in rural environment considerably more often than the control persons, but this was not associated with a higher percentage of occupation in agriculture. There was also a higher percentage of university graduates in the ALS group. Patients with bulbar onset were considerably more often born in an urban environment as compared to spinal onset. Apart from education and environment, ALS phenotypes did not differ in any investigated environmental or life-style factor. The rate of disease progression was not influenced by any of the investigated environmental and life-style factors. The present study could not identify any dietary habit, smoking, physical activity, occupational factor as well as toxic influences as risk factor or protective factor for onset or progression of ALS. Living in rural environment and higher education might be associated with higher incidence of ALS.

ALS is the most common adult onset motor neuron disease. Recent findings suggest that ALS not only causes motor neuron death but rather represents a systemic disease which may also involve cognition, the extrapyramidal and sensory nervous system, among others (1-4). Pathogenesis of ALS is still not fully understood. A multifactorial etiology including genetic factors, oxidative stress, altered RNA metabolism and many others is presumed. The phenotype of ALS-patients differs substantially regarding body region of symptom onset (bulbar, cervical, lumbar), involvement of upper and lower motor neurons, progression rate or variety and severity of additional symptoms as dementia (5,6). Given these remarkable clinical heterogeneities between ALS patients, the question arises whether certain environmental and lifestyle factors could have an impact on the disease phenotype or whether there are specific risk factors for subgroups of patients. For example, several studies showed that specific comorbidities occur in ALS patients in different frequencies as compared to the normal population and can possibly also influence the disease course (7-9). According to the current literature, cardiovascular risk factors may have a protective impact whereas there is an ongoing discussion whether high levels of physical activity could be a negative factor (1,10-17). However, a definite environmental risk factor has not been identified so far.

In the present study we aimed to investigate whether distinct factors of environment e.g. birth and living in rural or urban areas or toxic influences and lifestyle e.g. smoking, diet, level of education, physical activity during leisure or at work of ALS patients can influence disease onset and progression and whether specific risk factors for the development of distinct phenotypes can be identified.

## MATERIALS AND METHODS

We analyzed a cohort of 117 ALS patients who have been treated as either inpatients or outpatients in our university hospital. All patients with ALS treated at Hannover Medical School in the period between January 2016 and February 2017 were asked and included after written informed consent. In addition, 93 controls were included into the study (patients’ spouses, hospital staff, other acquaintances). Data were collected by a personal interview developed by the ONWeBDUALS consortium, a group of European ALS researchers, based on input from an international survey among ALS experts worldwide (18).

The ALS patient group was characterized regarding gender, onset, age, disease duration and disease severity (Table 1).

We compared ALS patients and controls regarding physical activity, dietary habits, smoking, residential environment, potentially toxic environmental factors and profession before and throughout the course of disease. Physical activity was graded as “intense physical exercise > 1 year”, within this category either as “150min/week moderate aerobic activity” or “75min/week vigorous aerobic activity” versus “mild physical exercise”. These graduations should allow to investigate the possible impact of physical activity on onset or progression of the disease more precisely, e.g. to identify distinct sports or exercise habits as risk or protective factors. Occupational physical activity was classified as low (mostly sedentary), mean and high levels. Special dietary habits such as vegetarian, vegan, gluten-free and high protein diet were recorded. Smoking habits were classified as current smokers, non-smokers and ex-smokers. Regarding residential environment, birth place and urban/ rural living during the last five years before disease onset and more than five years before disease onset was assessed. A distinction was made between villages (<1000 inhabitants), country towns (1000-5000 inhabitants), small towns (5000 - 20000 inhabitants), middle towns (20000 - 100000 inhabitants) and large towns (>100000 inhabitants). Living near possibly toxic influences (sewage plant, landfills, waste incinerators, sources of EM fields as high voltage transmission lines or radar) was recorded.

**Table 1:** Characterization of the ALS patient cohort.

Characterization of patient cohort	ALS patients (n = 117)
Onset (spinal: bulbar), N (%)	96: 21 (82%: 18%)
UMN dominant: LMN dominant: UMN/LMN equally, N (%)	14: 83 :19 (12%: 71%: 16%)
Disease duration, median (Q25, Q75)	17 months (11 months; 25 months)
ALSFRS-R, median (Q25, Q75)	40 (35.25; 43)
ALSFRS-R decline per month, progression rate, mean (sd)	0,91 (0,89)

ALSFRS-R: Amyotrophic Lateral Sclerosis Functional Rating Scale - Revised, sd: standard deviation, Q: Quartile

Regarding the level of education, data of ALS patients were also compared with data from the German population obtained via the federal statistical office (19). In addition, sub-groups of distinct phenotypes of ALS patients (region of onset, extent of UMN/LMN involvement) were analyzed for differences concerning the named parameters.

We also studied the influence of these factors on the progression rate of the disease. Progression rate was defined as decrease of ALSFRS-R per month. Data for progression rate estimation was available for 58 of the 117 included ALS patients.

The assumption of a normal distribution was checked by boxplot graphics. Normally distributed variables were described using mean and standard deviation and compared using the two-sided t-test for independent samples (two groups) or using a univariate analysis of variance (ANOVA, more than two groups). Metric of non-normally distributed variables were described using median and quartiles (25% and 75%) and compared using the Mann-Whitney-U-Test (two groups) or using the Kruskal-Wallis-Test (more than two groups). Categorical variables were described using absolute and relative frequencies and compared using the Chi-Squared-Test or Fisher’s exact test (for cell frequencies < 5). To identify the influence of several factors on progression rate we used ANOVA. First all factors were analyzed separately by simple ANOVA. Parameters with presumably relevant influence (p < 0.2) were then analyzed together by multiple ANOVA. Using backward selection (exclusion limit: p = 0.05) relevant factors were finally identified. The percentage of university graduated persons in the ALS groups was compared to the national average in Germany using the exact binomial test for one proportion.

Because of the inflation of the alpha error due to multiple testing in the same sample, the collected p-values were assessed descriptively. The term “significant” was avoided.

Statistical analyses were conducted using SPSS V. 19 (SPSS, Chicago, IL).

**Figure 1.**
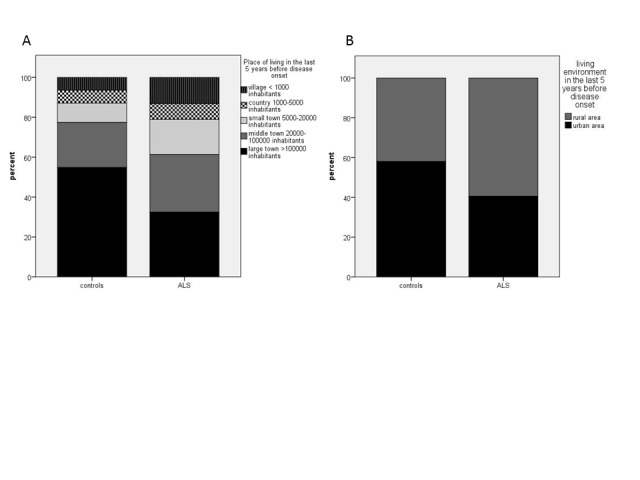
Place of living of ALS-patients and controls. ALS-patients relevantly more frequently lived in smaller towns (p = 0.021) (A) and rural areas (p = 0.013) (B).

### RESULTS

#### Comparison of ALS patients and controls

Disease specific variables are described in Table 1 and all results of the comparison of ALS patients and controls are shown in Table 2. There was no considerable difference between ALS patients and controls regarding age (p= 0.128) but there was a substantially higher proportion of men in the ALS-patient group than in the control group (p = 0.001). ALS Patients and controls did not differ regarding smoking and diet in our cohort. Special dietary habits (vegetarian, vegan, etc.) and distinct toxic influences (living near sewage plant, waste incinerators etc.) were only sporadically reported in both groups so that statistically relevant differences could not be identified. Also, the extent of physical training and occupational physical activity was not different between ALS patients and controls. Regarding physical activity, there was a tendency towards an increased extent of moderate training in ALS patients while controls tended to more frequently undergo training with higher intensity (p=0.081). Patients with regular physical exercise and/or more intense occupational physical activity did not show earlier disease onset.

**Figure 2.**
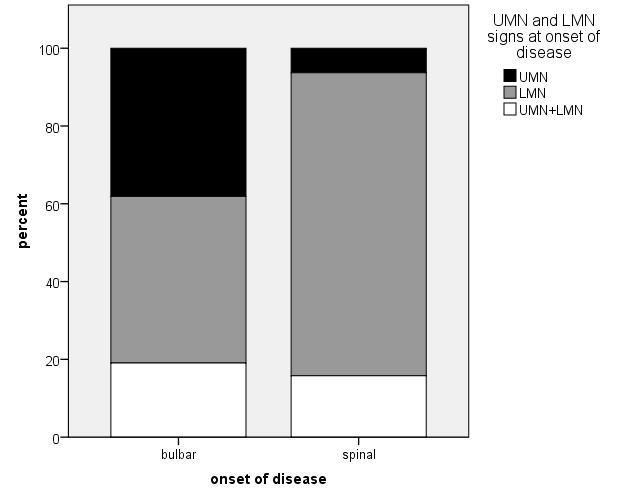
Patients with bulbar onset showed UMN involvement more frequently than patients with spinal onset (p<0.001).

**Table 2:** Comparison of patients and ALS-patients (93 vs 117).

variable	value	Controls	ALS	p-value
Age		58.68 (sd = 8.88)	60.79 (sd = 11.23)	0.13
Gender	female	54 (58.1)	42 (35.9)	0.0014
	male	39 (41.9)	75 (64.1)	
Occupational physical activity in the last 5 years before disease onset	low	18 (30.0)	16 (21.3)	0.1283
	mean	37 (61.7)	44 (58.7)	
	high	5 (8.3)	15 (20.0)	
Occupational physical activity more than 5 years before disease onset	low	21 (23.3)	24 (21.6)	0.7620
	mean	54 (60.0)	64 (57.7)	
	high	15 (16.7)	23 (20.7)	
dietary habits	none	79 (85.9)	103 (91.2)	0.5828
	vegetarian	6 (6.5)	5 (4.4)	
	Gluten free	1 (1.1)	0 (0.0)	
	Low carb	5 (5.4)	5 (4.4)	
	Lactose free	1 (1.1)	0 (0.0)	
Region of birth	rural	31 (33.3)	52 (47.3)	0.0441
	urban	62 (66.7)	58 (52.7)	
Place of birth	village (<1000 inhabitants)	13 (14.0)	23 (20.2)	0.1754
	country town (1000-5000 inhabitants)	6 (6.5)	9 (7.9)	
	small town (5000 - 20000 inhabitants)	7 (7.5)	18 (15.8)	
	middle towns (20000 - 100000 inhabitants)	26 (28.0)	23 (20.2)	
	large town (>100000 inhabitants)	41 (44.1)	41 (36.0)	
Region of living more than 5 years before disease onset	rural	34 (36.6)	61 (55.0)	0.0087
	urban	59 (63.4)	50 (45.0)	
Region of living in the last 5 years before disease onset	rural	39 (41.9)	66 (59.5)	0.0126
	urban	54 (58.1)	45 (40.5)	
Toxic influence in the last 5 years before disease onset	none	74 (79.6)	93 (82.3)	0.6628
	Sewage plant	6 (6.5)	6 (5.3)	
	Landfills	3 (3.2)	5 (4.4)	
	Waste incinerators	1 (1.1)	3 (2.7)	
	EM fields	7 (7.5)	6 (5.3)	
	Chemical plant	2 (2.2)	0 (0.0)	
Toxic influence more than 5 years before disease onset	none	79 (84.9)	92 (81.4)	0.7588
	Sewage plant	3 (3.2)	4 (3.5)	
	Landfills	2 (2.2)	3 (2.7)	
	Waste incinerators	2 (2.2)	3 (2.7)	
	EM fields	4 (4.3)	9 (8.0)	
	Chemical plant	1 (1.1)	2 (1.8)	
	Coal-burning power plant	2 (2.2)	0 (0.0)	
Place of living in the last 5 years before disease onset	village (<1000 inhabitants)	6 (6.5)	15 (13.2)	0.0214
	country town (1000-5000 inhabitants)	6 (6.5)	9 (7.9)	
	small town (5000 - 20000 inhabitants)	9 (9.7)	20 (17.5)	
	middle towns (20000 - 100000 inhabitants)	21 (22.6)	33 (28.9)	
	large town (>100000 inhabitants)	51 (54.8)	37 (32.5)	
Place of living more than 5 years before disease onset	village (<1000 inhabitants)	7 (7.5)	17 (14.9)	0.0024
	country town (1000-5000 inhabitants)	5 (5.4)	9 (7.9)	
	small town (5000 - 20000 inhabitants)	5 (5.4)	17 (14.9)	
	middle towns (20000 - 100000 inhabitants)	21 (22.6)	34 (29.8)	
	large town (>100000 inhabitants)	55 (59.1)	37 (32.5)	
Smoking habits	non-smokers	41 (44.1)	52 (46.0)	0.5596
	Ex-smokers	38 (40.9)	39 (34.5)	
	Current smokers	14 (15.1)	22 (19.5)	
Regular physical exercise	no	26 (28.0)	25 (22.1)	0.3344
	yes	67 (72.0)	88 (77.9)	
Physical exercise differentiated	No sport	10 (10.8)	12 (10.7)	0.0806
	Intense, 150min/week moderate aerobic activity	41 (44.1)	68 (60.7)	
	Intense, 75min/week vigorous aerobic activity	31 (33.3)	22 (19.6)	
	Mild physical exercise	11 (11.8)	10 (8.9)	

Absolute numbers of the study groups, percentages in brackets. Relevant p-values (< 0.2) are shown in bold.

ALS patients considerably more often than control patients lived in smaller towns and rural environment (p= 0.021 and p=0.013) (Fig. 1A and 1B, Table 2). The place of living was recorded for the last five years before disease onset and also for time period longer than five years before disease onset. There were no relevant differences between these two-time periods (not shown).

Due to inclusion of personal acquaintances into the control group, the percentage of university graduated persons might be erroneously high. For this parameter we therefore compared the ALS group with age-matched information from the federal statistical office (19). This analysis showed that there was a higher percentage of university graduates in the ALS group than in the general population regarding the age groups 30-39, >65 and by tendency 40-49 years (Table 3). Interestingly, university graduated patients had a tendency towards an earlier onset of disease (54.9 years (sd 13.32) vs. 59.4 years (sd 10.31), p= 0.069). Despite the potentially biased composition of our control group regarding occupation our data indicate, that ALS patients did not have more frequent occupations with moderate or higher physical activity or that there was an accumulation of special occupations in the ALS-group e.g. agriculture, contact with animals or electricity.

**Table 3:** Percentages of university graduates in our ALS patient group compared with the national average 2016 in Germany according to information of the federal statistical office.

age	Percentage of university graduates (%) ALS patients (absolute proportion of group)	Percentage of university graduates (%) National average 2016	p-value
30-39	100% (3/3)	27%	0.02
40-49	35.3% (6/17)	21%	0.15
50-64	14.0% (6/43)	18%	0.69
>65	22.4% (11/49)	12%	0.04

www.destatis.de/DE/ZahlenFakten/GesellschaftStaat/BildungForschungKultur/Bildungsstand/Aktuell.html

### Comparison of ALS phenotypes

**Figure 3.**
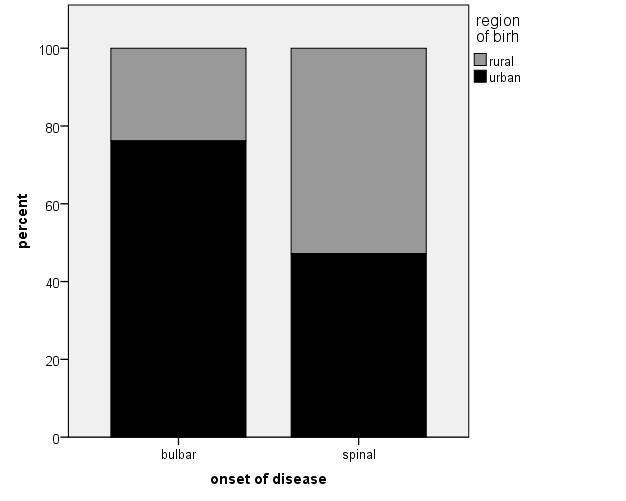
Bulbar onset patients relevantly more often than spinal onset patients were born in an urban environment (p = 0.017).

Within the ALS patients’ group, we also investigated different phenotypes such as patients with bulbar and spinal onset as well as patients with predominant upper motor n euron (UMN) or lower motor neuron (LMN) symptoms or with equal UMN and LMN symptoms. All results of these comparisons are displayed in table 4 and 5. Bulbar onset patients had shorter disease duration compared with spinal onset patients (p=0.013). Furthermore, patients with bulbar onset more frequently had UMN involvement than patients with spinal onset (p<0.001) (Fig. 2, Table 4). Patients with bulbar vs. spinal onset did not differ regarding smoking habits, physical activity in leisure time or occupation, education or living environment (). However, ALS patients with bulbar onset were considerably more often born in an urban environment compared to spinal onset patients (p= 0.017) (Fig. 3, Table 4). This result was independent of age, which did not differ relevantly between urban and rural born patients. Beside the more frequent spinal onset in LMN dominant patients, patients with LMN and UMN dominant phenotypes did not differ in any investigated environmental factor (Table 5).

The ALS-patients of our group have been tested for mutations in the SOD1 and C9orf72 genes. Two patients carried SOD1 mutations and seven patients C9orf72 mutations. The comparison of the seven C9orf72 patients with the other ALS-patients showed a relevant result regarding place of birth as six of the C9orf72 patient were born in a rural area (p=0.047).

**Table 4:** Comparison of ALS-patients with bulbar and spinal onset (21 vs 96).

variable	value	bulbar	spinal	p-value
age		61.19 (sd = 11.97)	60.70 (sd = 11.14)	0.8566
Gender	female	11 (52.4)	31 (32.3)	0.0821
	male	10 (47.6)	65 (67.7)	
UMN and LMN symptoms	predominant upper motor neuron (UMN)	8 (38.1)	6 (6.3)	<0.001
	predominant lower motor neuron (LMN)	9 (42.9)	74 (77.9)	
	equal UMN and LMN symptoms	4 (19)	15 (15.8)	
Level of education	No university graduate	17 (85.0)	69 (75.0)	0.3988
	University graduate	3 (15.0)	23 (25.0)	
Occupational physical activity in the last 5 years before disease onset	low	3 (21.4)	13 (21.3)	1.0000
	mean	8 (57.1)	36 (59.0)	
	high	3 (21.4)	12 (19.7)	
Occupational physical activity more than 5 years before disease onset	low	5 (25.0)	19 (20.9)	0.8405
	mean	12 (60.0)	52 (57.1)	
	high	3 (15.0)	20 (22.0)	
Dietary habits	none	20 (95.2)	83 (90.2)	0.8251
	vegetarian	1 (4.8)	4 (4.3)	
	Low carb	0 (0.0)	5 (5.4)	
Region of birth	rural	5 (23.8)	47 (52.8)	0.0167
	Urban	16 (76.2)	42 (47.2)	
Place of birth	village (<1000 inhabitants)	3 (14.3)	20 (21.5)	0.2890
	country town (1000-5000 inhabitants)	1 (4.8)	8 (8.6)	
	small town (5000 - 20000 inhabitants)	1 (4.8)	17 (18.3)	
	middle towns (20000 - 100000 inhabitants)	7 (33.3)	16 (17.2)	
	large town (>100000 inhabitants)	9 (42.9)	32 (34.4)	
Region of living more than 5 years before disease onset	rural	11 (52.4)	50 (55.6)	0.7923
	urban	10 (47.6)	40 (44.4)	
Region of living in the last 5 years before disease onset	rural	11 (52.4)	55 (61.1)	0.4631
	urban	10 (47.6)	35 (38.9)	
Toxic influence in the last 5 years before disease onset	none	19 (90.5)	74 (80.4)	0.9067
	Sewage plant	1 (4.8)	5 (5.4)	
	Landfills	1 (4.8)	4 (4.3)	
	Waste incinerators	0 (0.0)	3 (3.3)	
	EM fields	0 (0.0)	6 (6.5)	
Toxic influence more than 5 years before disease onset	none	20 (95.2)	72 (78.3)	0.4817
	Sewage plant	0 (0.0)	4 (4.3)	
	Landfills	1 (4.8)	2 (2.2)	
	Waste incinerators	0 (0.0)	3 (3.3)	
	EM fields	0 (0.0)	9 (9.8)	
	Chemical plant	0 (0.0)	2 (2.2)	
Place of living in the last 5 years before disease onset	village (<1000 inhabitants)	2 (9.5)	13 (14.0)	0.4116
	country town (1000-5000 inhabitants)	2 (9.5)	7 (7.5)	
	small town (5000 - 20000 inhabitants)	5 (23.8)	15 (16.1)	
	middle towns (20000 - 100000 inhabitants)	3 (14.3)	30 (32.3)	
	large town (>100000 inhabitants)	9 (42.9)	28 (30.1)	
Place of living more than 5 years before disease onset	village (<1000 inhabitants)	2 (9.5)	15 (16.1)	0.2627
	country town (1000-5000 inhabitants)	2 (9.5)	7 (7.5)	
	small town (5000 - 20000 inhabitants)	5 (23.8)	12 (12.9)	
	middle towns (20000 - 100000 inhabitants)	3 (14.3)	31 (33.3)	
	large town (>100000 inhabitants)	9 (42.9)	28 (30.1)	
Smoking habits	non-smokers	10 (47.6)	42 (45.7)	0.9021
	Ex-smokers	8 (38.1)	31 (33.7)	
	Current smokers	3 (14.3)	19 (20.7)	
Regular physical exercise	no	7 (33.3)	18 (19.6)	0.1702
	yes	14 (66.7)	74 (80.4)	
Physical exercise differentiated	No sport	5 (25.0)	7 (7.6)	0.0949
	Intense, 150min/week moderate aerobic activity	9 (45.0)	59 (64.1)	
	Intense, 75min/week vigorous aerobic activity	5 (25.0)	17 (18.5)	
	Mild physical exercise	1 (5.0)	9 (9.8)	

Absolute numbers of the study groups, percentages in brackets. Relevant p-values (< 0.2) are shown in bold.

### Influence on disease progression

**Figure 4.**
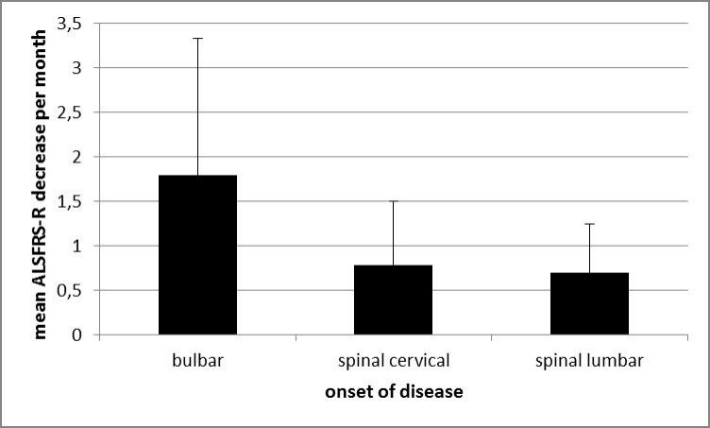
Bulbar onset was associated with faster disease progression (p=0.001).

Neither physical activity at leisure nor occupational physical activity had an impact on the progression rate of the disease. Neither did smoking and diet or living- and birth- environment, level of education and the presence of SOD1/C9orf72 mutations influence the progression rate. Only bulbar onset (Fig. 4) and right-handedness were associated with faster disease progression (p=0.001 and p=0.027). However, as we could only include three left handed patients the latter result was not considered any further.

### DISCUSSION

To investigate possible risk or protective factors in lifestyle and environment we analyzed a cohort of 117 ALS patients and 93 controls recruited at Hannover Medical School for the ONWeBDUALS register (18).

Physical activity has been intensively discussed as a potential risk factor for developing ALS (1,13). An association between ALS and professional soccer or American football playing has been described (11,20,21). However, patients of our group did not report increased physical activity at leisure or occupation compared with control patients. These results are in keeping with recent and rather consistent (level A) evidence from the literature which concludes that physical activity rather is not a risk factor for ALS (10,14,15,22,23). The relations between professional athleticism and ALS could also be due to other unknown environmental or lifestyle factors like frequent traumas or drug intake (10,16). Recently it was even suggested that physical activity may eventually be a protective factor (12). Within our ALS patient group, the extent of reported physical activity had no influence on the disease progression. We could therefore not support previous findings that physical exercises might have a neuroprotective effect (22).

Our data showed no influence of smoking on the incidence of ALS, also not in bulbar cases as previously described (24-27). Neither did smoking accelerate disease progression in our study population, as suggested by another previous study (28). Our results are in line with several other studies showing no relation between smoking and ALS (29, 30).

We further could not find an increased exposure to toxic influences in the ALS population. ALS patients lived relevantly more often in rural environment than controls. Living in rural environment has been previously described as a risk factor for ALS in several studies (31-33) but the literature is controversial (34-36). A potential reason might be increased exposure to agricultural chemicals (i.e. herbicides, insecticides, fungicides). Some studies found an increased number of ALS patients with an occupation in agriculture and therefore concluded that not rural residence itself but agricultural activities could influence the risk of ALS (29, 37-39). However, this finding is also controversial (35, 40-42) and we could not find a cluster of patients with agriculture occupation in our ALS group, also not in the bulbar subgroup. Some studies identified an association of other occupations with ALS e.g. in the building and construction sector, veterinarians, hairdressers, electricians, those exposed to magnetic fields and some others (24, 42-45) but a reliable proof that would confirm a possible connection between any occupation and ALS is still lacking and there were no clusters of specific occupations in our cohort. In this context it is interesting that six of seven C9orf72 patients were born in a rural area. One can speculate whether this mutation is more frequent in rural areas in Germany.

There were more university graduates in the ALS group than one might expect compared to the general German population. Moreover, patients with a university degree, interestingly, by trend develop ALS at younger age. Together this could hint to a higher risk of ALS for people with higher education. Previous studies have described both an association between high (31) and low (29,32) levels of education and ALS, others did not find any association between education and ALS (46). The fact that individuals with ALS who did not seek medical advice or who died before diagnosis cannot be included in any study needs to be addressed in this context. Patients with higher education are more likely to notice a symptom earlier and to visit a tertiary ALS center. In Parkinson’s disease, door-to-door studies showed that almost 40% of patients remain undiagnosed (38, 47). Therefore, larger and thoroughly conducted studies are necessary before drawing any conclusions.

Disease progression was not influenced by diet, smoking, physical activity at leisure or work, environment of birth and living place, toxic influences or educational level.

Regarding the different phenotypes of ALS, bulbar onset was considerably more frequent in patients born in urban than in rural environment. This has not been described before and the reason remains unclear for now.

The present study has several limitations. With its retrospective, interview-based design there might be a potential recall bias even though patients were carefully instructed to focus on the presymptomatic stage when answering the questions regarding occupation, place of living and physical activity.

The recruitment of an appropriate control group for epidemiological questions presents some difficulties. As ALS is more frequent in men, including only the spouses of patients leads to a relevantly higher amount of women in the control group compared with the ALS patient group which was the case in our study population. Including hospital staff and personal acquaintances can also distort the control group with a greater extent of higher educated people as it might have happened in our control group. This bias can also influence other parameters beside education level such as living place or habits of physical activity and diet. On the other hand, if one intends to investigate epidemiological questions like education level or living environment, it is not possible to match the ALS and control group for these parameters as one cannot analyze their frequencies afterwards. Therefore, it is hardly possible to imitate the random compilation of an ALS patient group, if it is random at all. Perhaps recruitment of multiple groups of controls could address this problem in future case control studies (47).

Another limitation is that the number of participants in the present study is only moderate and did not allow for investigation of all known phenotypes as the subgroups would have been too small for statistical analysis. Data for progression rate only were only available for 50% of the ALS group.

**Table 5:** Comparison of ALS-patients with predominant upper motor neuron (UMN) or lower motor neuron (LMN) symptoms or with equal UMN and LMN symptoms (14 vs 83 vs 19).

variable	value	predominant upper motor neuron	predominant lower motor neuron	equal UMN and LMN symptoms	p-value
Age		60.29 (sd = 9.77)	61.99 (sd = 10.51)	57.47 (sd = 13.18)	0.2599
Gender	female	9 (64.3)	26 (31.3)	7 (36.8)	0.0597
	male	5 (35.7)	57 (68.7)	12 (63.2)	
Onset	bulbar	8 (38.1)	9 (42.9)	4 (19)	<0.001
	spinal	6 (6.3)	74 (77.9)	15 (15.8)	
Level of education	No university graduate	13 (92.9)	60 (76.9)	13 (68.4)	0.2692
	University graduate	1 (7.1)	18 (23.1)	6 (31.6)	
Occupational physical activity in the last 5 years before disease onset	low	1 (10.0)	14 (26.4)	1 (9.1)	0.4383
	mean	7 (70.0)	27 (50.9)	9 (81.8)	
	high	2 (20.0)	12 (22.6)	1 (9.1)	
Occupational physical activity more than 5 years before disease onset	low	4 (28.6)	17 (22.1)	3 (15.8)	0.2071
	mean	8 (57.1)	40 (51.9)	15 (78.9)	
	high	2 (14.3)	20 (26.0)	1 (5.3)	
Dietary habits	none	13 (92.9)	74 (93.7)	15 (78.9)	0.1488
	vegetarian	0 (0.0)	3 (3.8)	2 (10.5)	
	Low carb	1 (7.1)	2 (2.5)	2 (10.5)	
Region of birth	rural	5 (38.5)	39 (50.6)	8 (42.1)	0.6214
	Urban	8 (61.5)	38 (49.4)	11 (57.9)	
Place of birth	village (<1000 inhabitants)	2 (14.3)	16 (20.0)	5 (26.3)	0.8052
	country town (1000-5000 inhabitants)	2 (14.3)	6 (7.5)	1 (5.3)	
	small town (5000 - 20000 inhabitants)	3 (21.4)	13 (16.3)	2 (10.5)	
	middle towns (20000 - 100000 inhabitants)	1 (7.1)	19 (23.8)	3 (15.8)	
	large town (>100000 inhabitants)	6 (42.9)	26 (32.5)	8 (42.1)	
Region of living more than 5 years before disease onset	rural	8 (57.1)	43 (55.8)	9 (47.4)	0.7846
	urban	6 (42.9)	34 (44.2)	10 (52.6)	
Region of living in the last 5 years before disease onset	rural	9 (64.3)	44 (57.1)	12 (63.2)	0.8158
	urban	5 (35.7)	33 (42.9)	7 (36.8)	
Toxic influence in the last 5 years before disease onset	none	12 (85.7)	63 (79.7)	17 (89.5)	0.9868
	Sewage plant	1 (7.1)	4 (5.1)	1 (5.3)	
	Landfills	0 (0.0)	5 (6.3)	0 (0.0)	
	Waste incinerators	0 (0.0)	3 (3.8)	0 (0.0)	
	EM fields	1 (7.1)	4 (5.1)	1 (5.3)	
Toxic influence more than 5 years before disease onset	none	12 (85.7)	62 (78.5)	17 (89.5)	0.9886
	Sewage plant	1 (7.1)	3 (3.8)	0 (0.0)	
	Landfills	0 (0.0)	3 (3.8)	0 (0.0)	
	Waste incinerators	0 (0.0)	3 (3.8)	0 (0.0)	
	EM fields	1 (7.1)	6 (7.6)	2 (10.5)	
	Chemical plant	0 (0.0)	2 (2.5)	0 (0.0)	
Place of living in the last 5 years before disease onset	village (<1000 inhabitants)	1 (7.1)	11 (13.8)	3 (15.8)	0.5935
	country town (1000-5000 inhabitants)	1 (7.1)	5 (6.3)	3 (15.8)	
	small town (5000 - 20000 inhabitants)	5 (35.7)	13 (16.3)	2 (10.5)	
	middle towns (20000 - 100000 inhabitants)	2 (14.3)	25 (31.3)	5 (26.3)	
	large town (>100000 inhabitants)	5 (35.7)	26 (32.5)	6 (31.6)	
Place of living more than 5 years before disease onset	village (<1000 inhabitants)	2 (14.3)	13 (16.3)	2 (10.5)	0.3135
	country town (1000-5000 inhabitants)	1 (7.1)	6 (7.5)	2 (10.5)	
	small town (5000 - 20000 inhabitants)	5 (35.7)	11 (13.8)	1 (5.3)	
	middle towns (20000 - 100000 inhabitants)	1 (7.1)	24 (30.0)	8 (42.1)	
	large town (>100000 inhabitants)	5 (35.7)	26 (32.5)	6 (31.6)	
Smoking habits	non-smokers	4 (28.6)	39 (48.8)	9 (50.0)	0.3673
	Ex-smokers	8 (57.1)	24 (30.0)	7 (38.9)	
	Current smokers	2 (14.3)	17 (21.3)	2 (11.1)	
Regular physical exercise	no	4 (28.6)	17 (21.5)	4 (21.1)	0.7764
	yes	10 (71.4)	62 (78.5)	15 (78.9)	
Physical exercise differentiated	No sport	4 (28.6)	6 (7.7)	2 (10.5)	0.4372
	Intense, 150min/week moderate aerobic activity	7 (50.0)	50 (64.1)	11 (57.9)	
	Intense, 75min/week vigorous aerobic activity	2 (14.3)	14 (17.9)	5 (26.3)	
	Mild physical exercise	1 (7.1)	8 (10.3)	1 (5.3)	

Absolute numbers of the study groups, percentages in brackets. Relevant p-values (p < 0.2) are shown in bold.

It has to be noted that the results should be interpreted with caution and need to be verified independently. Despite these limitations, our present investigation is one of the few studies that addressed numerous environment and life style factors in relation to different phenotypes of ALS as previously suggested (48) and also analyzed a possible impact on the disease course. Therefore, it provides valuable information and should trigger similar but larger longitudinal multicenter studies.

### Conclusion

The variability in phenotypes and progression rate strongly suggests an impact of occupational, environmental or lifestyle hazards in ALS. The present study could not identify any risk or protective role for dietary habits, smoking or physical activity. Living in rural environment might be associated with higher incidence of ALS. Patients with higher education levels appear to have a higher risk of developing ALS or at least were more frequently diagnosed with ALS and included in studies. Regarding different phenotypes, bulbar onset was considerably more frequent in patients born in urban than in rural environment. Disease progression was not influenced by any of the investigated environmental and life-style factors. Spinal onset patients had a slower progression. Larger multicenter studies are necessary to reassess the results of our study.
